# Childhood Habits: Ignorance is not Bliss— A Prevalence Study

**DOI:** 10.5005/jp-journals-10005-1037

**Published:** 2009-04-26

**Authors:** Amitha M Hegde, Arun M Xavier

**Affiliations:** 1 Professor and Head, Department of Pedodontics and Preventive Children Dentistry, AB Shetty Memorial Institute of Dental Sciences, Mangalore, Karnataka, India; 2 Postgraduate Student, Department of Pedodontics and Preventive Children Dentistry, AB Shetty Memorial Institute of Dental Sciences, Mangalore, Karnataka, India

**Keywords:** Children, lip biting, malocclusion, nail biting, prevalence, sucking.

## Abstract

Underneath the ignorance of adverse oral habits and general health practices in children proved to fetch harm, lies the basis and the motive in carrying out this study. This survey screened a total of 2636 children between the age group 4 and 15 years, residing in areas in South Kanara district and the prevalence of the adverse habits were assessed using a questionnaire and clinical examination. 526 students (19.95%) were found to be victims of adverse oral habits with nail biting being the most prevalent habit and bruxism, the least. Though the overall percentage of knowledge on basic body cleanliness was good, a small fraction weren’t aware, thus posing the necessity of educating children right from home and school and thus ensuring that the health of ones child is safe and secure.

## INTRODUCTION

A habit is a sign of lack of harmony between an individual and his environment. The American Academy of Pediatric Dentistry (AAPD) recognizes that an infant’s, child’s, or adolescent’s well-being can be affected by oral habits creating a need for effective individual management of the same.^1^

Adverse oral habits as thumb sucking, tongue thrusting, lip and cheek biting may produce harmful effects on the development of maxillofacial complex, facial hyper divergency resulting in anterior open bites^2^,^3^ and posterior cross bites in children.^4^,^5^ The effects of habitual nail biting include oral carriage of enterobacteriaceae,^6^ small fractures at the edges of incisors, gingivitis and orthodontic complications. 7

Prevalence studies in parts of India by Kharbanda OP et al,^8^ Munshi AK and Shetty^9^ depict 25.5% and 29.7% of children respectively as victims of such oral habits. In view of the aforementioned complications, there arises a need to highlight the current ill-practices in the society and encourage the cultivation of healthful habits and lifestyle.

A concept of health care, viz., self care, refers to those activities undertaken by the person themselves in promoting their own health, preventing diseases and illness.^10^ Motivation of the population in regard to maintenance of good hygiene should be emphasized. The paucity of studies on the subject necessitates an assessment of the prevalence of such habits and education of the common masses.

## DESIGN

This cross-sectional study was conducted in a total population of 2636 children, aged between 4 and 15 years from schools situated in rural, semi urban and urban areas in South Kanara District, Karnataka.

The oral screening was carried out by trained personnel from the Department of Pedodontics and Preventive Children’s dentistry, A.B. Shetty Memorial Institute of Dental Sciences, Mangalore, using clinical mouth mirrors and probes with emphasis on adverse oral habits.

**Table 1:** Table1: Prevalence of various adverse oral habits and associated pathology in children

			*Number of*		*Percent*		*Male*		*Female*		*Associated pathology*		
			*students*		*(%)*		*(%)*		*(%)*		*n*		*(%)*
	Habits												
	Nail biting		232		44.11		20.34		23.76		69		29.74
	Tongue thrusting		177		33.65		16.35		17.30		112		63.28
	Lip biting		53		10.07		3.42		6.65		41		77.36
	Pencil biting		49		9.32		5.13		4.18		27		55.10
	Thumb sucking		10		1.9		1.14		0.57		08		80
	Bruxism		05		0.95		0.57		0.38		05		100
	Total		526										

An interviewer - administered questionnaire was used to gather the data on the prevalence of habits, both oral and general, parental attitudes, knowledge and self awareness. A Health-educational talk delivery followed the examination with a computer aided media and study models.

## RESULTS

Of the screened 2636 student population, 65% were males and 35% were females. It was found that among the sum total of students screened, 19.95% (526 students) were victimized by adverse oral habits, where girls (53.04%) had a major share over boys (46.05%).

Assessing the prevalence of these deleterious oral habits (Table 1), it was found that 44.11% (232 students) had a characteristic nail biting habit. Following the nail biting habit, 33.65% (177 students) had tongue thrusting, 10.07% (53 students) lip biting, 9.32% (49 students) pencil biting, 1.9% (10 students) thumb sucking and 0.95% (5 students) bruxism habits respectively. 69 chronic nail biters and 27 pencil biting children depicted incisal wear. 112 students had proclination of teeth with central diastema due to the tongue thrusting habit. Severe malocclusion and facial hyperdivergency was noticed in 8 children with thumb sucking. The 5 students with the bruxism habit had generalized incisal/occlusal wear with associated temporomandibular joint disorders (TMDs).

**Fig. 1.**
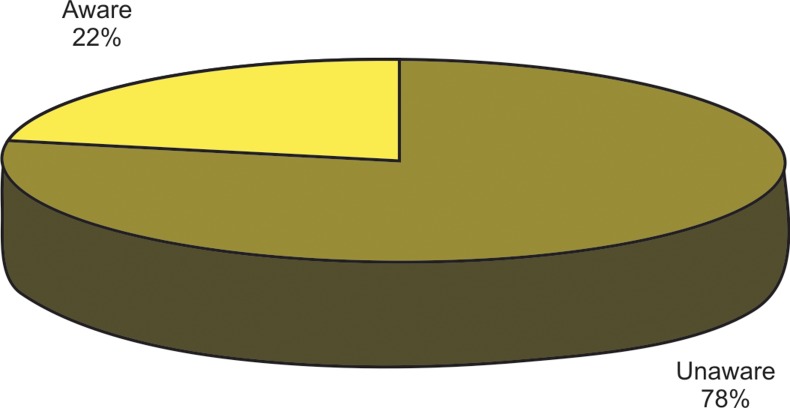
Illustrates the awareness among the school children regarding the harmful effects that habit they possessed could fetch

Nearly 22% of the students were guided by their parents regarding the harmful effects of their oral habits, but the astonishing aspect is the continuation of the same. 77.8% of the students did not know the ill effects the habit could fetch and so relentlessly continued their performance (Fig. 1).

In the programme organized, the health talk provided to these children after the oral screening created awareness regarding the habit, whereby questions were asked pertaining to the sequelae and necessity of early interception of adverse oral habits. More than 70% of the audience comprehended the talked about habits and their sequelae per se.

**Fig. 2.**
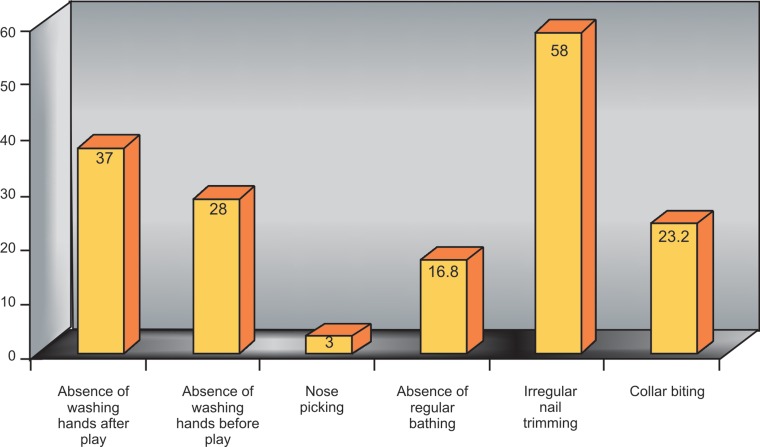
Prevalent adverse general habits among the screened children

The questionnaire that included parameters on the awareness and maintenance of general health (Fig. 2) affirmed that 63% students washed hands after playing in a playground. Only 72% students used to wash hands before any meal. 3% of students had nose picking, only 83.2% children bathed regularly, 45.2% students did not have the habit of using a handkerchief to wipe their face and used their shirt collar and sleeves to do so. Only 42% students timely trimmed their nails. This points out the ignorance of these children towards the maintenance of cleanliness. Awareness regarding the first Aid Kit was also assessed, and only 38.11% of the screened children knew regarding its role in emergencies. 16.23% of children did not know the importance of being clean and hence the necessity of helping these children at the earliest.

## DISCUSSION

This study conducted in rural, semi urban and urban school population depicts a high percentage of 77% children unaware of the ill effects of oral habits. As high a figure of 22.2% inspite of being aware of the effects continue the habits, symbolic of negligence and ignorance. Studies done previously on the oral habits showed a high prevalence of 25.5%^8^ and 29.7%.^9^ Prevalence in our study appears to be 19.95% which may be attributed to increasing awareness among the masses. Due to better developing medical facilities with each advancing day, common people have a better access and hence better knowledge and attitude development regarding their health. Partly, a smaller sample size when compared to previous studies may be considered in our study. Any variability could also be due to examiner discrepancy as more than 2 personnel conducted the examination. Findings of the present study are in disagreement with the observations by Guaba et al^11^ who found only 3% of children in the age range of 6-15 years to demonstrate an oral habit.

Our study affirms a higher prevalence of 44.11% of nail biting among the screened population accompanied by worn out incisal edges and orthodontic complications as open bite. Following it, was the tongue thrusting habit at a prevalence rate of 33.65%, thumb sucking and accompanying proclination of teeth with open bite and cross bites. Behavioral problems in school children are common due to excessive stress, competitiveness, high parental expectations and consequent anxiety.^12^ Agarwal et al^13^ has indirectly related nail biting, thumb sucking, etc. to indicate highly stressful and anxiety-related behavior. Lip biting presented as lip contusions and proclination followed by pencil biting. Bruxism was represented by worn incisal edges and dentinal hypersensitivity. A sexual variation appears with a higher prevalence in the females unlike a study witnessing a similar prevalence in both the sexes.^8^

No less prevalent were faulty general practices like washing hands before a meal, after playing in a playground, nose picking associated with nail biting, nail trimming and bathing regularly. 10% of the nail biters had characteristic stains on their shirt collars, when probed into, unveiled a strange habit of collar biting by these children. Maintenance of hygiene and practices mentioned are inter-related to the oral carriage of enterobacteriaceae causing a plethora of problems.^6^ When questioned about awareness regarding first aid, 38.11% were not aware of the basic medical help in untoward situations.

To summarize the scenario, there is a paucity of surveys generating a felt need to conduct more such surveys to serve the purpose of educating the common masses and to create more awareness towards a healthy lifestyle and the basic medical amenities available. A healthy mouth is the index of a healthy body and healthy body is an abode to a healthy mind.

## CONCLUSION

- 19.95% children had adverse oral habits. The most prevalent oral habit was Nail biting (44.11%) followed by tongue thrusting (33.65%) and the least prevalent was bruxism (0.95%). Another interesting habit noticed was that of collar biting.
- 77.8% students were unaware of the potential hazards that the adverse oral habit could pose on one’s well being.
- Prevalence of adverse general health practices ranged from 0.3 to 45%, with 16.23% children unaware of the importance of basic body cleanliness.
